# P-1413. Association of Electronic Patient Portal Use and HIV Viral Suppression and Retention in Care among People Living with HIV at an Urban Academic Medical Center

**DOI:** 10.1093/ofid/ofae631.1588

**Published:** 2025-01-29

**Authors:** Daniela Zimmer, Neda Laiteerapong, Raj Shetty, Jessica Ridgway

**Affiliations:** University of Chicago Medicine, Chicago, Illinois; University of Chicago Medicine, Chicago, Illinois; University of Chicago, Chicago, Illinois; University of Chicago Medicine, Chicago, Illinois

## Abstract

**Background:**

Electronic patient portals are secure websites that give patients access to health information and allow for secure messaging with providers. Portal use has been associated with improved patient engagement in care, better patient-provider communication, and improved health outcomes in non-HIV patient populations, but portal use is under-studied among PLWH. We sought to assess the relationship between patient portal (MyChart) utilization and HIV-related outcomes among PLWH.

**Methods:**

We collected electronic health record data for PLWH who attended an HIV care visit at an urban academic medical center over 2 years, including demographics, laboratory results, medical encounters, and MyChart status. We compared the demographic characteristics of PLWH with and without an active MyChart account using chi-square. We used logistic regression to assess if MyChart status was associated with outcomes of viral suppression (< 200 copies/mL), retention in care (using the six-month gap definition, i.e., PLWH who attended an HIV care appointment in the prior 6 months are retained), and CD4 count >200 cells/mm^3^, after controlling for age, race, and sex.

**Results:**

Among 785 PLWH, the mean age was 47 years (SD=14.9), 70% were male, and 83% were Black/African-American. Black PLWH were significantly less likely than White PLWH to have an active MyChart account (83.6% (543/649) vs. 95.8% (91/95), p< 0.01). PLWH with an active MyChart had increased odds of HIV viral suppression (Odds Ratio [OR]: 2.02, 95% CI [1.3 - 3.7]); retention in care (OR: 2.2 95% CI [1.4 -3.4]); and CD4 >200 (OR: 3.3, 95% CI [1.8 to 6.0].

**Conclusion:**

MyChart utilization at the urban academic HIV clinic is associated with positive health outcomes. However, Black PLWH are less likely to utilize MyChart than White PLWH. Additional work is needed to increase MyChart access for non-white patients PLWH, to decrease the digital divide, improve engagement in care and other health outcomes.

**Disclosures:**

**Jessica Ridgway, MD**, Gilead Sciences: Expert Testimony

